# Peri-implant bone loss in platform-switched vs conventional implants: A radiographic analysis

**DOI:** 10.6026/973206300213249

**Published:** 2025-09-30

**Authors:** Prashant A. Karni, Ravindra Babu Lakkaraju, Sachin Yadav, Yasasvi Kalluri, Mutthineni Ramesh Babu, Prateek Tripathy, Rahul Tiwari

**Affiliations:** 1 Department of Prosthodontics and Crown and Bridge, KAHER’s KLE VK Institute of Dental Sciences, Belagavi, Karnataka, India; 2 Department of Dental Surgery, Government Medical College, Quthbullapur, Medchal-Malkajgiri, Telangana, India; 3 Department of Dentistry, Government Medical College, Alwar, Rajasthan, India; 4 Department of Periodontics and Implantology, Private Practitioner, Kakarla Dental Hospital, Tirupati, Andhra Pradesh, India; 5 Department of Periodontics, Mamata Dental College, Khammam, Telangana, India; 6 Department of Oral and Maxillofacial Surgery, Hi-Tech Dental College and Hospital, Bhubaneswar, Odisha, India; 7 Department of Oral and Maxillofacial Surgery, RKDF Dental College and Research Centre, Sarvepalli Radhakrishnan University, Bhopal, Madhya Pradesh, India

**Keywords:** Dental implants, dental implant-abutment design, alveolar bone loss, radiography, dental, retrospective studies

## Abstract

Platform-switching (PS) reduces peri-implant marginal bone loss (MBL) compared with conventional platform-matched (PM). Hence, we conducted a single-center retrospective radiographic study comparing 12-month MBL around PS and PM bone-level implants restored with fixed prostheses. Standardized periapical radiographs were calibrated to implant thread pitch and analyzed by blinded examiners. Among 128 implants (PS, n=64; PM, n=64), mean 12-month MBL was 0.28 ± 0.24 mm for PS vs 0.44 ± 0.31 mm for PM; the adjusted mean difference was -0.16 mm (95% CI -0.24 to -0.08; p=0.001). Thus, PS also showed a lower (non-significant) proportion with MBL ≥ 1.5 mm.

## Background:

Preserving crestal bone is critical for long-term implant success, as early marginal bone loss (MBL) may compromise stability and esthetics [1]. Platform switching (PS), achieved by placing a narrower abutment on a wider implant platform, is proposed to shift the implant-abutment micro gap away from bone, thereby reducing inflammatory effects and limiting vertical remodeling compared with platform-matched (PM) designs [2, 3]. Clinical studies suggest PS leads to modest but significant reductions in early MBL, with differences ranging from 0.2 to 0.4 mm at one year [4, 5]. Soft-tissue thickness, abutment height and placement depth may further influence outcomes, with thicker mucosa and longer abutments supporting more favorable bone stability [6]. Radiographic evaluations over several years have confirmed that PS implants show slower bone loss or even stabilization compared to PM designs [7, 8]. Therefore, it is of interest to assess through radiographic analysis, whether PS implants reduce 12-month MBL compared with PM implants.

## Materials and Methods:

This retrospective cohort analyzed records from a specialty practice. Adults restored with bone-level implants supporting single crowns or short fixed partial dentures were included if functionally loaded for ≥12 months and had standardized periapical radiographs at delivery (T0) and 12 ± 2 months (T12). Exclusions were immediate failures, graft complications, uncontrolled systemic disease, heavy smoking (>10/day), or prior peri-implantitis therapy. The platform-switched (PS) group received narrower abutments on wider implant platforms, while platform-matched (PM) used matching abutments; multiple brands were accepted if workflows were comparable. Radiographs were taken with paralleling technique, calibrated to implant dimensions, and bone loss measured from implant-abutment junction to first bone contact mesially and distally. Two blinded examiners assessed independently, with consensus for discrepancies. Covariates included age, sex, jaw, site, implant diameter, placement depth, history of periodontitis, and smoking. Analyses used mixed-effects linear and logistic models with patient-level clustering; significance set at p<0.05.

## Results:

Baseline characteristics were comparable between groups (Table 1 - see PDF). Mean patient age was 54 years, with similar sex distribution, jaw location, implant diameter, placement depth and history of periodontitis, confirming balanced cohorts. At 12 months, platform-switched (PS) implants demonstrated significantly less marginal bone loss (MBL) than platform-matched (PM) implants (0.28 ± 0.24 mm vs 0.44 ± 0.31 mm). The adjusted mean difference was -0.16 mm (95% CI: -0.24 to -0.08; p = 0.001), indicating a clear benefit of PS in reducing early crestal bone remodeling (Table 2 - see PDF). Clinically relevant thresholds showed fewer implants with MBL ≥1.5 mm in the PS group (3.1%) than in PM (10.9%), though this difference did not reach statistical significance (p = 0.08). Biological complications, such as bleeding on probing, occurred less frequently in PS (9.4%) than PM (15.6%), and technical complications (screw loosening) were rare in both groups. Importantly, prosthesis survival was 100% in both groups, demonstrating equivalent short-term functional outcomes (Table 2 - see PDF).

## Discussion:

Our results are coherent with a recent split-mouth clinical trial directly comparing PS and PM connections over three years, which reported higher initial and cumulative MBL for PM (butt-joint) interfaces despite otherwise similar restorative protocols [9, 10]. Although our cohort included both crestal and sub crestal placements, interaction testing did not show effect modification by placement depth-consistent with randomized data indicating that, within conical PS systems, 1- to 2-mm sub crestal positioning does not significantly change early MBL provided soft-tissue parameters are respected [10]. From a prosthetic standpoint, splinting strategy and framework design can influence load distribution and hygiene access, thereby affecting bone behavior; large retrospective series of splinted fixed bridges show small but measurable MBL that is more sensitive to plaque control and emergence profile than to the mere presence of splinting per se [11]. In our adjusted models, a history of periodontitis and larger implant diameter were associated with higher MBL, echoing broader evidence that patient-level susceptibility and restorative geometry contribute meaningfully to early changes [11]. Guidance from a comprehensive prosthetic design systematic review emphasizes that connection type, abutment protocol, and restoration contours jointly shape MBL outcomes; the review concluded that tissue-level and optimized bone-level designs can both perform well when prosthetic variables are planned to preserve the supracrestal tissue dimension [12]. Our findings support prioritizing restorative space for biologic width-achieved either by PS or by ensuring adequate vertical soft tissue and abutment height-to mitigate early bone remodeling [12]. Evidence emerging from a randomized split-mouth trial with multiyear follow-up suggests PS advantages can persist irrespective of external-hex or Morse-taper macro-connection, with PM showing greater crestal remodeling in otherwise matched scenarios [13].

Still, the magnitude of benefit is generally small at one year, reinforcing that PS should be viewed as one component of a multifactorial strategy rather than a stand-alone solution [13]. Meta-analytic data on abutment height provide a biological rationale for our observed association: longer transmucosal components help establish a stable supracrestal tissue height, which correlates with less early MBL around bone-level/PS implants [14]. Complementarily, minimizing abutment disconnections ("one-time abutment" approach) may curtail repeated disruption of the soft-tissue seal and modestly improve bone preservation, particularly during the early remodeling window [15]. Clinically, the ≈0.16 mm difference we observed is unlikely to be discernible in the short term for most patients; however, in risk-enriched settings (thin tissues, short vertical restorative space, or reduced distances between restoration margins and bone crest), PS may provide additional biological latitude to maintain the crestal seal [9, 12]. The non-significant reduction in the proportion of implants with MBL ≥ 1.5 mm in PS hints at potential threshold effects that larger or longer studies could clarify [10, 11]. Strengths include blinded radiographic assessment with high reliability and mixed-effects modeling accounting for clustering. Limitations include retrospective design, brand heterogeneity, and restriction to 12-month outcomes; cone-beam evaluation of three-dimensional remodeling was not performed, and plaque indices were not consistently documented. Future randomized research integrating standardized emergence profiles, abutment heights, and restoration margin-to-crest distances could delineate the relative contributions of each factor within PS and PM contexts.

## Conclusion:

Platform-switching was associated with a small but statistically significant reduction in 12-month marginal bone loss compared with platform-matched connections. The clinical relevance is context-dependent and should be integrated with soft-tissue thickness, abutment height, and restorative contours. Careful prosthetic planning may outweigh connection type alone.
